# Productive, internal organ and intestinal histomorphological characteristics of broiler chickens in response to dietary rapeseed meal: A meta‐analysis

**DOI:** 10.1111/jpn.14040

**Published:** 2024-09-30

**Authors:** Freddy Manyeula, Nthabiseng Amanda Sebola, Monnye Mabelebele

**Affiliations:** ^1^ Department of Agriculture and Animal Health College of Agriculture and Environmental Sciences, University of South Africa Pretoria South Africa

**Keywords:** broiler chickens, carcass traits, feed intake, gut health, rapeseed meal, weight gain

## Abstract

The use of rapeseed as a source of protein in broiler chicken diets has been highlighted. However, there are inconsistent findings on the performance data of broiler chickens fed rapeseed meal (RSM). Therefore, this meta‐analysis aimed to resolve the inconsistent findings on the effect of RSM on growth performance, carcass characteristics, internal organs, and intestinal histomorphology of broiler chickens, identify knowledge gaps and create new insights using published data. Fourteen studies on the topic were identified via a systematic search performed on bibliographic databases, and the data generated was analysed using OpenMEE software. A random‐effects model was used, and effect sizes were presented as standardised mean difference (SMD) at a 95% confidence interval (CI). Sources of heterogeneity were evaluated using broiler strains, inclusion levels, processing methods, rearing phases and sex as moderators. In comparison with the controls, the results showed that RSM decreased feed intake (SMD = −0.29; 95% Cl: −0.41, −0.18; *p* < 0.001), average daily gain (SMD = −0.48; 95% Cl: −0.63, −0.32; *p* < 0.001), and liver weight (SMD = 1.24; 95% Cl: 0.78, 1.71; *p* < 0.001), but had no effect on feed conversion ratio (SMD = 0.10; 95% Cl: −0.05, 0.23; *p* = 0.19). Likewise, broiler chickens fed RSM had significantly reduced carcass yield, weights of thigh, abdominal fat and heart when compared with the control. Results indicate that duodenum villus height (DVH) and jejunum villus height (JVH)/crypt depth (CD) ratios were improved in broiler chickens fed RSM. Meta‐regression revealed that the analysed moderators are significant predictors of feed intake, average daily gain and feed conversion ratio in broiler chickens. In conclusion, dietary RSM negatively influenced growth performance, liver weight and carcass characteristics in broiler chickens, but improved aspects of intestinal histomorphology traits. Therefore, innovative research on processing methods that will improve the feeding value of rapeseed meal in broiler chickens is recommended.

## 1 INTRODUCTION

Poultry meat has been projected to increase globally to 152 metric tonnes over the projection period, thus accounting for 52% of the other meat consumed (OECD/FAO, 2021). This meat is regarded as healthier than red meat since it has less cholesterol and fat content (Farrell, 2013). The OECD/Food Agriculture Organisation of the United Nations, (2014) highlighted that poultry meat expands faster, with the expectation that it will increase by 1.8% per year from 2015 to 2024. The expansion of this poultry meat industry is directly influenced by the high cost of soybean meal, which constitutes about 66% of the protein in broiler diets (Veldkamp & Bosch, 2016). This is aggravated by the competition for soybean meal between chickens, humans and the industry. Thus, this calls for the development of more cost‐effective alternatives to soybean in broiler chicken diets.

One such alternative to soybean meal is rape seed meal (RSM), a by‐product of oil extraction from *Brassica spp.*, that has limited human food and industrial uses and is rich in protein and essential amino acids (Yahbi et al., 2024; Yang et al., 2022). However, the major limitation to the use of RSM in poultry feed is its high content of antinutritional factors (ANFs), including glucosinolates, tannins and phytic acids. These ANFs have been reported to cause liver haemorrhagic and necrosis, resulting in nutrient absorption (Yang et al., 2022). However, numerous processing methods have been devised to detoxify these ANFs in RSM (Hu et al., 2016; Rozan et al., 1996). Several authors found inconsistent results in broiler chickens fed diets containing differently processed RSM (Ashayerizadeh et al., 2018; Tuunainen et al., 2016; Wu et al., 2020a, 2020b). These inconsistent results could be attributed to differences in broiler strain, sex, inclusion levels, processing methods and rearing phases reported by Ogbuewu and Mbajiorgu (2022) to influence broiler chicken performance.

The use of meta‐analytical methods to resolve inconsistent findings across studies that addressed the same objective and increase statistical power has been documented in the literature (Ogbuewu & Mbajiorgu, 2022). Currently, there is little or no published meta‐analysis that assesses the performance data of broilers fed RSM. Hence, this paper aimed to evaluate the meta‐analytic effect of RSM on growth performances, internal organ, carcass characteristics and intestinal histomorphology of broiler chickens.

## 2 MATERIAL AND METHODS

### 2.1 Literature search and selection criteria

**Figure 1:**
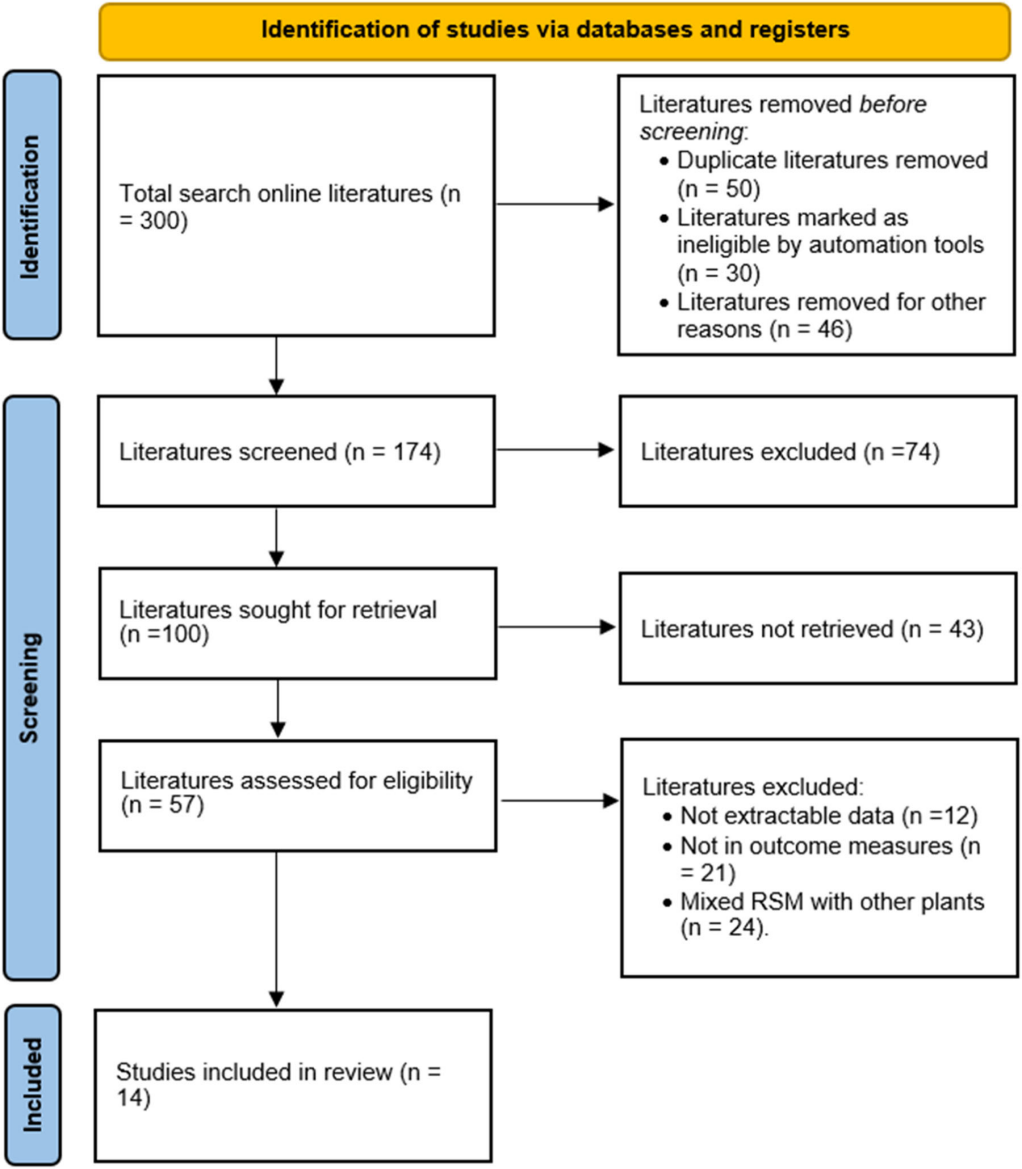
Literature search and selection process following the PRISMA procedure.

Google Scholar (https://scholar.google.com), Scopus (https://www.scopus.com), Web of sciences (https://www.webofscience.com) and PubMed (https://pubmed.ncbi.nlm.nih.gov) databases were searched for published articles on the impacts of RSM on growth performance, internal organs, carcass characteristics and intestinal histomorphology of broiler chickens using the following keywords: ‘broiler chickens’, ‘growth performance’, ‘internal organs’, ‘carcass characteristics’, ‘intestinal histology’ and ‘rapeseed meal’. A total of 300 studies were identified of which 14 met the inclusion criteria following the Preferred Reporting Items for Systematic Reviews and Meta‐analyses (PRISMA) flow diagram displayed in Figure 1. The included articles satisfied the following inclusion criteria; the title was related to the topic. Included studies measured atleast of the response parameters of interest (growth performance, internal organs, carcass characteristics and intestinal histomorphology) in broiler chickens with their measures of variance. Review studies on the topic were excluded from the meta‐analysis; studies on the topic not reported in broiler chickens were also excluded. Duplicated studies were removed.

### 2.2 Data extraction and analysis

Data on the surname of the first author, year of publication, study country (United States, Iran, Canada, Brazil, Egypt, Korea, South Africa and Australia), study continents (Europe, North America, Asia and Oceania), number of birds used for the experiment, number of treatments and moderator variables (strain [Ross, Heritage, Cobb, Leghorn and Arbor Acres], processing methods [fermented and unfermented], sex [male or female], inclusion levels [0%–10%, 11%–20%, 21%–30%, and 31%–40%] and production stage [starter, finisher and overall]) were retrieved from the 14 articles that met the inclusion criteria as presented in Table 1. All analyses were done in OpenMEE software. Effect sizes were pooled and expressed as standardised mean differences (SMD) at 95% confidence interval (CI). SMD was said to be significant when zero is not included in 95% CI. Heterogeneity was evaluated using Q‐statistic and *I* ^2^‐statistic (Higgins & Thompson, 2002) and was considered significant at 5%. The quantity of *I* ^2^ accounted by the moderators was determined through meta‐regression analysis. The effect of moderate on growth traits in broilers fed RSM was determined via subgroup analysis. Sensitivity analysis was done to determine the number of negative trials required to convert a statistically significant combined difference into a nonsignificant difference using a standard method (Lean et al., 2009). Publication bias was evaluated via Nfs and funnel plot methods. However, Jennions et al. (2013), revealed that outcomes of meta‐analysis can be deemed robust, notwithstanding the existence of publication bias when Nfs is higher than [5 × (*n*) + 10], where *n* = number of comparisons.

**Table 1:** Classification of studies included in this meta‐analysis.

						Explanatory variables				
S/N	References	Country	Continent	NBT	NT	Strain	Sex	IL	PM	Outcomes^a^
1	Karunajeewa et al. (1990)	Australia	Oceania	400	5	Tegel	M	0‐30	U	1, 2, 3, 13, 16
2	Ramesh et al. (2006)	India	Asia	624	4	Cobb	MS	0‐30	U	1, 2, 3, 13
3	Taraz et al. (2006)	Iran	Asia	200	4	Arian	M	0‐40	U	1, 2, 3, 4, 13, 16
4	Montazer‐Sadegh et al. (2008)	Iran	Asia	400	4	Ross	NR	0‐16	U	1, 2, 3, 4, 13, 16
5	Toghyani et al. (2009)	Iran	Asia	384	8	Ross	F	0‐25	E	13,1, 2, 3, 13, 16
6	Chiang et al. (2010)	China	Asia	168	3	Arbor Acres	M	NR	F	1, 2, 3, 6, 7, 8, 9, 10, 11, 12, 15
7	Xu et al. (2011)	China	Asia	399	7	Ross	M	0‐20	F	1, 2, 3, 6, 7, 8, 9, 10, 11, 12
8	Ashayerizadeh et al. (2018)	Iran	Asia	300	4	Cobb	M	0‐40	F	1, 2, 3, 4, 13, 14, 16
9	Biesek et al. (2020)	Poland	Europe	444	6	Ross	M	0‐30	U	1, 2, 3, 4, 5, 16, 14
10	Gao et al. (2020)	Poland	Europe	420	3	Ross	F	0‐15	F	1, 2, 3
11	Wu et al. (2022)	China	Asia	420	7	Arbor acres	M	0‐20	F	1, 2, 3, 4,5, 6, 7, 8, 12,14 15, 16
12	Pirgozliev et al. (2022)	United Kingdom	Europe	144	3	Ross	M	0‐28	E	1, 2, 3
13	Yadav et al. (2022)	United States	Europe	640	4	Cobb	M	0‐30	E	6, 7, 8, 9, 10, 11, 12, 15
14	Wiśniewska et al. (2023)	Poland	Europe	384	4	Ross	M	0‐15	E	1, 2, 3

Abbreviations: F, female; F, fermented; IL, inclusion level; M, male; MS, mixed sex; NA, North America; NBT, number of birds used for the study; NR, not reported; NT, number of treatments; PM, process methods; U, untreated.

^a^ Outcomes: 1 = feed intake; 2 = average daily gain; 3 = feed conversion ratio; 4 = abdominal fat; 5 = breast weight; 6 = duodenum crypt depth; 7 = duodenum villus height; 8 = duodenum villus height/crypt depth; 9 = ileum crypt depth; 10 = ileum villus height; 11 = jejunum crypt depth; 12 = jejunum villus depth; 13 = liver weight; 14 = thigh weight; 15 = ileum villus height/crypt depth; 16 = carcass yield.

## 3 RESULTS

### 3.1 Overview of studies used in the meta‐analysis

Table 1 depicts the features of 14 articles used in this meta‐analysis. The included studies were published between 1990 and 2023. The studies were done in seven countries (Australia, India, Iran, China, Poland, United Kingdom and United States) drawn from three continents (Oceania, Asia and Europe). Five thousand three hundred and seven broiler chickens were used in the experimental group, 1400 broiler chickens were used in the control group. Tegel, Cobb, Ross, Arial and Arbor acres strains aged 1–42 days fed RSM at an inclusion level of 0%–40% were used in this meta‐analysis.

### 3.2 Growth performance and carcass characteristics

The effects of dietary RSM on growth performance, internal organ and carcass characteristics of broiler chickens are presented in Table 2. Meta‐analysis results showed that broiler chickens fed RSM had significantly reduced feed intake (SMD = −0.29; *p* < 0.001; *I* ^2^ = 90%), ADG (SMD = −0.48; *p* < 0.001; *I* ^2^ = 94%), carcass yield (SMD = −0.15; *p* < 0.01; *I* ^2^ = 67%), thigh weights (SMD = −0.29; *p* < 0.03; *I* ^2^ = 82%), abdominal fat weight (SMD = −0.97; *p* < 0.001; *I* ^2^ = 97%), and heart weight (SMD = −2.01; *p* < 0.001; *I* ^2^ = 98%) compared to the controls. In contrast, RSM diets had no significant effect on FCR (SMD = 0.10; *p* = 0.19; *I* ^2^ = 94%) and breast weight (SMD = −0.29; *p* < 0.06; *I* ^2^ = 67%). However, broiler chickens fed RSM diets had heavier liver weight than the control (1.24; *p* < 0.001; *I* ^2^ = 98%).

**Table 2:** Growth performance, internal organ and carcass characteristics of broiler chickens fed RSM.

		95% CI				Heterogeneity			
Outcomes	SMD	Lower	Upper	SE	*p* Value	Q	*df*	*p* Value	*I* ^2^
Growth performance									
Feed intake	−0.29	−0.41	−0.18	0.06	<0.001	1129.7	110	<0.001	90
ADG	−0.48	−0.63	−0.32	0.08	<0.001	1540.8	96	<0.001	94
FCR	0.10	−0.05	0.23	0.08	0.191	1607.0	102	<0.001	94
Carcass characteristics									
Carcass yield	−0.15	−0.23	−0.03	0.06	0.010	85.2	28	<0.001	67
Breast weight	−0.29	0.60	0.016	0.16	0.063	78.5	10	<0.001	87
Thigh weight	−0.29	−0.55	−0.02	0.13	0.032	57.1	10	<0.001	82
Abdominal fat	−0.97	−1.46	−0.48	0.25	<0.001	1178.5	29	<0.001	97
Liver weight	1.24	0.78	1.71	0.24	<0.001	710.8	24	<0.001	96
Heart weight	−2.01	−2.98	−1.16	0.47	<0.001	811.9	14	<0.001	98

Abbreviations: ADG, average daily weight; CI, confidence interval; *df*, degree of freedom; FCR, feed conversion ratio; *I* ^2^, inconsistency index; *p*, probability; Q, Cochran statistic; SE, standard error; SMD, standardised mean difference.

### 3.3 Intestinal histomorphology

Table 3 showed the influence of RSM diets on intestinal histomorphology of broiler chickens. In comparison with the controls, pooled estimation results revealed that broiler chickens fed RSM had improved duodenum villus height (SMD = 0.62; *p* < 0.001; *I* ^2^ = 93%), JVH (SMD = 0.55; *p* < 0.05; *I* ^2^ = 96%), and JVH/CD (SMD = 0.74; *p* < 0.004; *I* ^2^ = 96%). The results shows that broiler chickens fed RSM had comparable duodenum crypt depth (SMD = 0.15; *p* < 0.001; *I* ^2^ = 93%), DVH/CD (SMD = 0.44; *p* < 0.05; *I* ^2^ = 96%), ileum crypt depth (SMD = 0.22; *p* < 0.31; *I* ^2^ = 93%), ileum villus height (SMD = 0.10; *p* < 0.001; *I* ^2^ = 95%) and JCD (SMD = −0.26; *p* = 0.17; *I* ^2^ = 93%) to those fed control diet.

**Table 3:** Intestinal histomorphology of broiler chickens fed RSM‐based diets.

		95% CI				Heterogeneity			
Outcomes	SMD	Lower	Upper	SEM	*p* Value	Q	*df*	*p* Value	*I* ^2^
DVH	0.62	0.27	0.98	0.18	<0.001	248.29	17	<0.001	93
DCD	0.15	−0.31	0.62	0.23	0.520	421.79	17	<0.001	95
DVH/CD	0.44	−0.01	0.88	0.22	0.061	383.10	17	<0.001	95
ICD	0.22	−0.21	0.65	0.22	0.311	156.82	11	<0.001	93
IVH	0.10	−0.43	0.62	0.27	0.732	224.54	11	<0.001	95
JCD	−0.26	−0.62	0.11	0.19	0.172	264.27	17	<0.001	93
JVH	0.55	0.00	1.10	0.28	0.053	538.02	17	<0.001	96
JVH/CD	0.74	0.23	1.25	0.25	0.004	475.58	17	<0.001	96

Abbreviations: CI, confidence interval; DCD, duodenum crypt depth; *df*, degree of freedom; DVH, duodenum villus height; DVH/CD, duodenum villus height/crypt depth ratio; *I* ^2^, inconsistency index; ICD, ileum villus depth; IVH, ileum villus height; JCD, jejunum crypt depth; JVH, jejunum villus depth; JVH/CD, jejunum villus height/crypt depth ratio; *p*, probability; Q, Cochran statistic; SE, standard error; SMD, standardised mean difference.

### 3.4 Subgroup analyses and meta‐regression

Subgroup analysis results of the impact of studied moderators on feed intake (FI) in broiler chickens fed RSM‐based diets are shown in Table 4. Cobb and Arian strains offered diets with and without RSM had similar FI. In contrast, Ross, Abor Acres and Teyel strains fed RSM had significantly lower FI than the control. Broiler chickens that received RSM 1%–10% had similar FI with the controls. In comparison with the controls, broiler chickens fed diets containing RSM at 11–20, 21–30 and >31% had significantly lower FI. Similarly, broilers that received untreated RSM and RSM supplemented with emulsifier + enzyme consumed less feed than the controls. In converse, broiler chickens fed RSM diets supplemented with enzyme and emulsifier separately had comparable FI with the controls. Male broiler chickens fed RSM diets had significantly reduced FI compared to the control. Broiler chickens drawn from studies that used female and mixed sexed birds had comparable FI with the controls. Rearing phases affected FI in broiler chickens with birds on RSM diets having lower FI than the control.

**Table 4:** Subgroup analyses of the effect of covariates of RSM on feed intake of broiler chickens.

		Random effects			Heterogeneity	
Subgroup	Nc	SMD	95% CI	*p* Value	*I* ^2^ (%)	*p* Value
Strain						
Cobb	24	−0.22	−0.50, 0.07	0.140	92.80	<0.001
Ross	30	−0.21	−0.32, −0.10	<0.001	67.20	<0.001
Arbor Acres	33	−0.20	−0.39, −0.01	0.040	86.96	<0.001
Tegel	12	−1.46	−1.10, −0.96	<0.001	93.33	<0.001
Arian	12	0.17	−0.03, 0.36	0.090	57.09	<0.001
Inclusion						
1–10	53	−0.18	−0.30, 0.06	0.110	83.80	<0.001
11–20	36	−0.24	−0.41, −0.07	0.007	85.20	<0.001
21–30	17	−0.83	−1.42, −0.25	0.005	96.90	<0.001
>31	5	−0.18	−0.41, −0.04	0.010	36.40	<0.001
Processing methods						
Untreated	69	−0.41	−0.56, −0.25	<0.001	91.90	<0.001
Fermented	24	0.08	−0.03, −0.19	0.170	46.70	<0.001
Enzyme	14	−0.20	−0.52, 0.12	0.220	88.30	<0.001
Emulsifier	2	−0.56	−1.13, 0.02	0.060	87.30	0.05
Emulsifier + enzyme	2	−0.76	−1.47, −0.03	0.040	91.50	0.001
Rearing phases						
Starter	39	−0.40	−0.64, −0.17	<0.001	92.90	<0.001
Finisher	23	−0.41	−0.66, −0.17	0.001	89.30	<0.001
Overall	49	−0.14	−0.28, −0.003	0.050	85.60	<0.001
Sex						
Male	62	−0.41	−0.56, −0.23	<0.001	92.40	<0.001
Female	8	−0.03	−0.17, 0.11	0.690	0.00	0.92
Mixed	6	−0.15	−0.49, 0.18	0.370	93.00	<0.001
Unknown	21	−0.18	−0.27, −0.09	<0.001	19.70	<0.001

Abbreviations: CI, confidence interval; *I* ^2^, inconsistency index; Nc, number of comparisons; *p*, probability; SE, standard error; SMD, standardised mean difference.

Table 5 shows the results of the effect of studied moderators on ADG of broiler chickens fed RSM‐based diets. The results revealed that Ross and Cobb strains had similar ADG with the controls, but Arbor Acres, Tegel and Arian strains had significantly reduced ADG when compared to the controls. Broilers fed untreated RSM had lower ADG than the controls. In contrast, broiler chickens fed emulsified RSM had higher ADG than the control. However, those fed fermented, enzyme supplemented, and emulsifier + enzyme supplemented RSM did not differ from those offered control diets. Rearing phases affected ADG in broiler chickens offered RSM‐based diets. Male, female and mixed sexed broiler chickens fed RSM had significantly reduced ADG.

**Table 5:** Subgroup analyses of the effect of covariates on ADG of broiler chickens fed RSM‐based diets.

		Random effects			Heterogeneity	
Subgroup	Nc	SMD	95% CI	*p* Value	*I* ^2^ (%)	*p* Value
Strain						
Cobb	12	−0.28	−0.75, 0.18	0.240	94.7	<0.001
Ross	28	−0.01	−0.24, 0.23	0.120	92.4	<0.001
Arbor Acres	33	−0.49	−0.68, −0.31	<0.001	86.7	<0.001
Tegel	12	−1.23	−1.78, −0.78	<0.001	93.6	<0.001
Arian	12	−0.98	−1.56, −0.39	0.001	94.4	<0.001
Inclusion						
1–10	49	−0.09	−0.21, 0.03	0.120	80.9	<0.001
11–20	30	−0.44	−0.72, −0.17	0.002	93.04	<0.001
21–30	13	−1.50	−2.17, −0.83	<0.001	96.50	<0.001
>31	5	−1.94	−2.78, −1.11	<0.001	93.00	<0.001
Process method						
Untreated	63	−0.65	−0.88, −0.42	<0.001	95.00	<0.001
Fermented	24	−0.25	−0.43, 0.08	0.090	79.90	<0.001
Enzyme	6	−0.16	−0.57, 0.26	0.460	85.50	<0.001
Emulsifier	2	0.20	0.001, 0.40	0.050	0.00	0.440
Emulsifier + enzyme	2	0.47	0.27, 0.67	0.100	0.00	0.750
Rearing phase						
Starter	31	−0.48	−0.81, −0.14	0.006	95.80	<0.001
Finisher	23	−0.55	−0.80, −0.30	<0.001	89.20	<0.001
Overall	43	−0.44	−0.66, −0.22	<0.001	92.90	<0.001
Sex						
Male	62	−0.73	−0.93, −0.53	<0.001	94.00	<0.001
Female	8	−0.37	−0.57, −0.16	<0.001	52.80	0.040
Mixed	6	0.36	−0.08, −0.65	0.010	71.10	0.004
Unknown	21	−0.02	−0.31, 0.27	0.890	92.10	<0.001

Abbreviations: CI, confidence interval; *I* ^2^, inconsistency index; Nc, number of comparisons; *p*, probability; SE, standard error; SMD, standardised mean difference.

The subgroup results of FCR in broiler chickens fed RSM are showed in Table 6. Tegel strain had significantly improved FCR, while the Arian and Arbor acres strains had poor FCR when compared to the controls. However, Cobb and Ross had comparable FCR with the controls. Results showed that broiler chickens fed >31% RSM had poor FCR, whereas FCR was not affected in broiler chickens fed 1%–10%, 11%–20% and 21%–30% RSM. Broiler chickens fed untreated RSM had significantly poorer FCR than the control, but those fed fermented and enzyme supplemented RSM had similar FCR with the controls. In contrast, broiler chickens fed emulsified and emulsified + enzymes supplemented RSM had lower FCR than the controls. In addition, broiler chickens fed RSM during the overall production phase had poor FCR. However, FCR were not affected in broilers fed RSM during the starter and finisher production phases when compared to the controls. Males and female broiler chickens fed RSM diets had poor FCR, while mixed and unknown sex had similar FCR to the controls.

**Table 6:** Subgroup analyses of the effect of moderators on FCR in broiler chickens fed RSM diets.

		Random effects			Heterogeneity	
Subgroup	Nc	SMD	95% CI	*p* Value	*I* ^2^ (%)	*p* Value
Strain						
Cobb	18	0.21	−0.66, 0.25	0.370	96.1	<0.001
Ross	28	−0.22	−0.52, 0.07	0.140	95.0	<0.001
Arbor Acres	33	0.28	0.17, 0.39	<0.001	65.4	<0.001
Tegel	12	−0.20	−0.31, −0.08	0.001	5.64	0.390
Arian	12	1.14	0.65, 1.62	<0.060	91.8	<0.001
Inclusion						
1–10	51	−0.10	−0.27,0.07	0.240	90.4	<0.001
11–20	32	0.12	−0.14, 0.37	0.360	92.8	<0.001
21–30	15	0.23	−0.31, 0.78	0.400	96.2	<0.001
>31	5	1.73	0.92, 2.53	<0.001	93.0	<0.001
Process methods						
Untreated	63	0.18	0.13, 0.42	<0.001	94.0	<0.001
Fermented	24	0.27	−0.02, 0.38	0.070	70.3	<0.001
Enzyme	12	−0.27	−0.77, 0.22	0.270	94.8	<0.001
Emulsifier	2	−0.74	−1.44, −0.04	0.040	91.2	0.001
Emulsifier + enzyme	2	−1.43	−1.92, −0.94	<0.001	78.9	0.030
Rearing phase						
Starter	35	−0.15	−0.48, 0.18	0.370	96.1	<0.001
Finisher	23	−0.14	−0.01, 0.29	0.060	71.9	<0.001
Overall	45	0.27	0.08, 0.47	0.007	91.7	<0.001
Sex						
Male	62	0.28	0.09, 0.47	0.004	93.5	<0.001
Female	8	0.30	0.05, 0.55	0.020	68.4	0.002
Mixed	12	−0.56	−1.12, 0.01	0.060	95.9	<0.001
Unknown	21	−0.13	−0.42, 0.16	0.370	92.2	<0.001

Abbreviations: CI, confidence interval; *I* ^2^, inconsistency index; Nc, number of comparisons; *p*, probability; SE, standard error; SMD, standardised mean difference.

Table 7 showed meta‐regression analysis of the influence of moderators on growth performance data of broiler chickens fed RSM‐based diets. The results of this meta‐regression revealed that strain and inclusion levels had effects on FI, ADG and FCR. Likewise, processing methods have associations with FI (*p* = 0.030) and FCR (*p* = 0.010) whereas, sex is a predictor for both ADG (*p* < 0.001) and FCR (0.007).

**Table 7:** Meta‐regression of the associations between moderators and growth parameters.

Outcomes	Moderators	Intercept	Q_M_	Estimate	*df*	*p* Value	*R* ^2^ (%)
Feed intake	Strains	−0.21	55.37	0.29	4	<0.001	34.3
	Inclusion level	−0.23	12.0	0.41	3	0.007	7.45
	Process methods	−0.41	10.6	0.41	4	0.030	6.23
	Rearing phases	−0.15	4.00	0.43	2	0.140	2.11
	Sex	−0.41	4.46	0.44	3	0.210	1.42
ADG	Strains	−0.28	26.22	0.60	4	<0.001	20.15
	Inclusion level	−0.44	60.4	0.45	3	<0.001	39.45
	Process methods	−0.64	8.2	0.72	5	0.140	3.16
	Rearing phases	−0.45	0.21	0.76	2	0.900	0.00
	Sex	−0.73	18.6	0.63	3	<0.001	15.01
FCR	Strains	−0.21	31.8	0.57	4	<0.001	22.8
	Inclusion level	0.12	23.0	0.61	3	<0.001	17.10
	Process methods	0.18	12.9	0.67	4	0.010	8.87
	Rearing phases	0.28	5.00	0.71	2	0.080	3.46
	Sex	0.28	0.007	0.67	3	0.007	8.54

Abbreviations: ADG, average daily gain; *df*, degree of freedom; FCR, feed conversion ratio; Q_M_, coefficient of moderator; *R* ^2^, amount of heterogeneity explained by moderators.

### 3.5 Publication bias analysis

**Figure 2:**
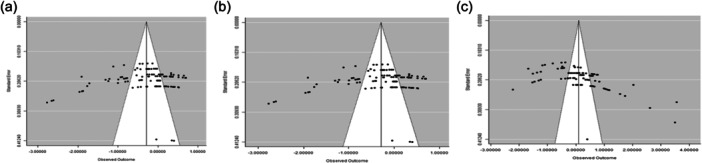
Funnel graphs of the effect of dietary RSM on (a) feed intake, (b) feed conversion ratio and (c) average daily gain in broiler chickens.

The results of publication bias analysis are presented in Figure 2 and reflects strong tendency for medium studies to be associated with greater positive effects for FI, ADG and FCR. The funnel graphs were asymmetrical indicating the presence of publication bias among the 14 articles. Rosenberg's fail‐safe number (Nfs) was further computed for evidence of publication bias. The fail‐safe numbers for the database for FI, ADG and FCR are 8997, 13702 and 72 which were 112.4, 195.7 and 0.9‐fold higher than the thresholds of 80 (5 × 14 + 10), 70 (5 × 12 + 10) and 80 (5 × 14 + 10), respectively, required for the mean effects size to be declared robust.

## 4 DISCUSSION

### 4.1 Growth performance

Growth performance and carcass traits are good indicators to assess diet quality. Feeding good quality diets to the broiler could promote nutrient digestibility, immune function, intestinal health, growth performance parameters and product quality. It is known that RSM contains glucosinolates, phenolic compounds and phytic acids, which interfere with nutrient utilisation. Glucosinolates are hydrolysed by myrosinase in the small intestine into glucose and unstable β‐aglycone, which spontaneously transforms into isothiocyanate, thiocyanate and nitriles. These by‐products inhibit iodine transportation into the thyroid follicular cells, thereby endangering health and hindering ADG (Tanii et al., 2004). Furthermore, lower feed intake in this meta‐analysis could be attributed to the presence of glucosinolates in RSM (Butler et al., 1984). These findings are consistent with the results of other researchers (Chiang et al., 2010; Montazer‐Sadegh et al., 2008), who reported lower feed intake in broilers fed RSM‐based diets. Additionally, phenolic compounds, and phytic acid form complexes with secretary enzymes, proteins, and minerals in the gastrointestinal tract, which in turn reduces nutrient utilisation, resulting in lower average daily gain in broiler chickens. The comparable FCR in broiler chickens fed diets with and without RSM suggests that feed utilisation was not adversely affected by RSM. This finding was in harmony with the results of Toghyani et al. (2009) and Biesek et al. (2020), who reported similar FCR on broiler fed RSM‐based diets.

### 4.2 Carcass characteristics

Diet quality could be measured by good carcass yield and well developed internal organs (Zhang et al., 2020). In this meta‐analysis, significantly reduced carcass yield, thigh weight, abdominal fat and heart weight may be attributed to the observed decline in FI. The decline in FI following the addition of RSM to the diets could be related to the ability of ANFs contained in RSM to alter the physicochemical properties of the diets. These results are in harmony with Taraz et al. (2006), who reported lower carcass and cut‐part weights in broiler chickens fed 23.61% RSM (100% replacement). These results are at variance with Fu et al. (2021), who reported similar carcass traits in animals other than broilers fed 16% RSM. The observed disparity might be attributed to differences in the breed of chicken and inclusion levels used. Breast muscle is the most economical valuable muscle in modern broiler production. However, in this meta‐analysis, the comparable breast weight of broiler chickens fed diets with and without RSM indicates that RSM supports breast muscle development in broiler chickens. These findings agree with the results of Taraz et al. (2006), who observed similar breast weight in broilers fed 0% and 25% RSM. Heavier liver in broilers fed RSM compared to those fed control could be increased activity of the liver to detoxify the ANFs present in RSM‐based diets (Payvastegan et al., 2017). A Similar observation has been reported in broiler chickens fed RSM‐based diets by Taraz et al. (2006).

### 4.3 Intestinal histomorphology

The development of the gastrointestinal tract could directly reflect the digestion and absorptive function of the gut. The crucial site of nutrients absorption in the intestine are the villi composed of the epithelial cells responsible for digestion and absorption of the nutrients. Also, the height of these villi and depth of the crypt, their ratio and thickness of the walls are good indicators for assessing the absorption capacity of the small intestine (Hernández et al., 2006; Montagne et al., 2004). Our meta‐analysis showed increased duodenum villus height, jejunum villus depth and villus height/crypt depth ratio in broiler chickens fed RSM‐based diets, indicating improved intestine function and health. The similar gut health of broiler chickens fed RSM‐based diets in the present meta‐analysis is could be attributed to the presence of fibre in RSM. Dietary fibre stimulates peristalsis movement which in turn enhance villus development. Zhang et al. (2023) reported that shorter villus indicates fewer mature chyme producing cell, while shallow crypt symbolises maturation of chyme producing cells. Wu et al. (2020b) and Konkol et al. (2024) reported improved absorptive capacity of laying hens and broiler chickens fed fermented RSM. On other hands, the comparable intestinal histomorphological traits in the present study imply similar absorptive capacity between broiler chickens fed RSM and control diets.

### 4.4 Subgroup analyses

#### 4.4.1 Strain

Subgroup analysis revealed that ability of broiler chickens to utilise RSM diets differs among the stains, which explains why Ross, Abor acres and Tegel strains had lower FI compared to Cobb and Arian. This result agrees with Ogbuewu et al. (2023) who reported that growth performance data of broiler chickens are influenced by chicken genetics. The lower ADG in Abor Acres, Tegel and Arian strains compared to Ross and Cobb strains are in harmony with Rondelli et al. (2003), who reported that chickens genetics affects growth indices. The poorer FCR in Arbor Acres and Arian fed RSM when compared to Tegel strain indicates better nutrients utilisation. This could be attributed to the ability of Tegel strain to handle the ANFs present in RSM diets. Choo et al. (2014) have reported significant differences in FCR among strains of broiler chickens.

#### 4.4.2 Inclusion levels

The comparable FI and ADG in broiler chickens fed RSM at 0%–10% inclusion levels indicates that the level that optimised ADG in broiler chickens. This observation agrees with McNeill et al. (2004), who reported similar FI and ADG in broiler chickens fed 0% and 10% RSM. Subgroup analysis showed decreased FI and ADG in feed RSM at a level beyond 11%, which could be due to imbalance in amino acid composition as found in broiler chickens fed 20%−25% RSM (Zhang et al., 2020). Broilers fed RSM at 0%–10%, 11%–20% and 21%–30% had comparable FCR with the control. This is in agreement with the findings of Mikulski et al. (2012) and Fu et al. (2021), who reported similar FCR in animals other than broiler chickens offered 16% and 18% RSM diets. Our results contradicted the previous reports of Olukosi et al. (2017) that 20% RSM yielded poor FCR in broiler chickens. The comparable FCR in the present study could be due to the high fibre level in RSM that might have stimulate villus development (Singh & Kim, 2021), resulting in improved feed utilisation. Conversely, higher RSM inclusion level (>31) resulted on poor FCR, indicating poor villus development (Jha & Mishra, 2021).

#### 4.4.3 Process methods

It is known that biological methods and biotransformation technology and their applications can be used to improve the nutritive quality of feedstuffs (Hu et al., 2016; Pal Vig & Walia, 2001; Rozan et al., 1996; Yang et al., 2022). In the present study, broilers fed untreated RSM had decreased FI and ADG, implying inability of broiler chickens to handle ANFs, including goitrin, isothyocyanates and glucobrassicin contained in raw RSM (Drewnowski & Gomez‐Carneros, 2000). This finding agrees with Wu et al. (2022), who found reduced FI and ADG in broiler fed 15% raw RSM. The comparable FI and ADG in broilers fed fermented, enzymes supplemented, emulsified and cocktails (enzymes and emulsified) RSM compared with the control indicate the effectiveness of these processing methods in reducing the ANFs to an acceptable levels. Fermentation is a great approach to enhance the taste and texture of diets by removing the bitter compounds in the substrate. The findings of this study is consistent with the results of Wu et al. (2022), who reported increased ADG in broiler chickens fed 15% fermented RSM. The poor FCR in broiler chickens fed untreated RSM may be linked with presence of glucosinolates, which corroborated the findings of others (Mikulski et al., 2012). In converse, broiler chickens fed emulsifier and emulsifier + enzyme supplemented RSM diets had improved FCR, implying the ability of these processes to ameliorate ANFs (glucosinolates), leading to improvement in nutrients utilisation. This observation corroborate with the findings of Drazbo et al. (2018), who found improved better growth performances in turkey fed fermented RSM than those offered unprocessed RSM.

#### 4.4.4 Rearing phases

Results indicate that rearing phases is a significant predictor of FI, FCR and ADG in broilers offered RSM and caused most of the variations in the measured outcomes. Results showed that broilers offered RSM at the starter, finisher and overall phases had reduced FI and ADG, indicating the inability of broiler chickens at different stages of growth to utilise the ANFs in raw RSM. These finding is in consistent with Zhu et al. (2019), who demonstrated a significantly reduced ADG in birds fed 20% RSM during the starter production phase. Broilers fed RSM diets for overall rearing periods had significantly poor FCR and similar pattern was found by Chiang et al. (2010) who reported poor FCR in broilers fed RSM at overall phases. In converse, comparable FCR observed in broilers chickens at the starter and finisher phases suggests that nutrients in the RSM were well converted to muscle by the broilers, a finding similar to Fu et al. (2021), who observed similar FCR in a geese aged 35–49 and 49–70 days.

#### 4.4.5 Sex

Subgroup result demonstrated that sex is a limiting factor in this study and can lead to varying growth performance results. The significant decreased FI (male and unknown) and ADG (male, female and mixed) in broiler observed in the current meta‐analysis suggests inability of these chicken to overcome ANFs. In contrast, poor FCR in male and females broiler chicken is an indication of consuming more feeds and convert them into less muscle. The nonsignificant difference in FI, ADG and FCR found in this analysis on different sexes suggests that RSM was utilised by different sexes of broilers.

### 4.5 Meta‐regression

The results showed evidence of significant relationships between broiler strain and inclusion level on FI, ADG and FCR. This finding agreed with study by Zhang et al. (2020), who reported different growth performance parameters among different broiler strains. Olusegun et al. (2020) also reported that strain and sex have a significant influence on growth performance parameters in broiler chickens. The inclusion level of RSM is an important factor that may affect growth performance in broiler chickens. Meta‐regression results revealed that processing method is a predictor FI and FCR in broiler chickens fed RSM‐based diets. A similar pattern has been reported in broiler chickens by Abdollahi et al. (2018). Meta‐regression results also revealed significant relationships between sex and aspects of outcome variables (FCR and ADG) which corroborates with the study by Benyi et al. (2015), who found relationship between sex and ADG on commercial strain chickens. Yang et al. (2015) also found association between growth parameters and inclusion levels in chickens.

## 5 CONCLUSION

The pooled results showed that diet containing RSM depressed FI and ADG but had no effect on FCR in broiler chicken. Furthermore, carcass yield, thigh weight, heart weight and abdominal fat weight were reduced by RSM diets. However, RSM diets increased breast and liver weights in broiler chickens. The pooled results indicated that RSM diets enhanced duodenum villus height and jejunum villi height/crypt depth ratio in broiler chickens. Subgroup analysis revealed that Cobb and Arian fed RSM at a level not more than 10% had improved growth performance data. In addition, Cobb and Arian strains utilised RSM‐based diets better than the Ross, Arbor Acres and Tegel strains. Broilers fed processed RSM had improved growth performance data than those fed raw RSM. Meta‐regression showed that studied moderators explained most of the sources of heterogeneity in the present meta‐analysis.

## AUTHOR CONTRIBUTIONS

**Freddy Manyeula**: Conceptualisation; data collection and analysis; writing original draft. **Nthabiseng Amanda Sebola**: Conceptualisation; writing review and editing. **Monnye Mabelebele**: Data collection and analysis; writing review and editing. All authors read and approved the final version.

## CONFLICT OF INTEREST STATEMENT

The authors declare no conflict of interest.

## Data Availability

Data will be made available on reasonable request.
